# Constructing disease-specific gene networks using pair-wise relevance metric: Application to colon cancer identifies interleukin 8, desmin and enolase 1 as the central elements

**DOI:** 10.1186/1752-0509-2-72

**Published:** 2008-08-10

**Authors:** Wei Jiang, Xia Li, Shaoqi Rao, Lihong Wang, Lei Du, Chuanxing Li, Chao Wu, Hongzhi Wang, Yadong Wang, Baofeng Yang

**Affiliations:** 1 College of Bioinformatics Science and Technology and Bio-pharmaceutical Key Laboratory of Heilongjiang Province, Harbin Medical University, Harbin 150081, PR China; 2 Department of Bioinformatics, Capital University of Medical Sciences, Beijing 100084, PR China; 3 Department of Medical Statistics and Epidemiology, School of Public Health, Sun Yat-Sen University, Guangzhou 510080, PR China; 4 Department of Molecular Cardiology, Cleveland Clinic Foundation, 9500 Euclid Avenue, Cleveland, Ohio 44195, USA; 5 Department of Computer Science, Harbin Institute of Technology, Harbin 150080, PR China

## Abstract

**Background:**

With the advance of large-scale omics technologies, it is now feasible to reversely engineer the underlying genetic networks that describe the complex interplays of molecular elements that lead to complex diseases. Current networking approaches are mainly focusing on building genetic networks at large without probing the interaction mechanisms specific to a physiological or disease condition. The aim of this study was thus to develop such a novel networking approach based on the relevance concept, which is ideal to reveal integrative effects of multiple genes in the underlying genetic circuit for complex diseases.

**Results:**

The approach started with identification of multiple disease pathways, called a gene forest, in which the genes extracted from the decision forest constructed by supervised learning of the genome-wide transcriptional profiles for patients and normal samples. Based on the newly identified disease mechanisms, a novel pair-wise relevance metric, adjusted frequency value, was used to define the degree of genetic relationship between two molecular determinants. We applied the proposed method to analyze a publicly available microarray dataset for colon cancer. The results demonstrated that the colon cancer-specific gene network captured the most important genetic interactions in several cellular processes, such as proliferation, apoptosis, differentiation, mitogenesis and immunity, which are known to be pivotal for tumourigenesis. Further analysis of the topological architecture of the network identified three known hub cancer genes [interleukin 8 (IL8) (*p *≈ 0), desmin (DES) (*p *= 2.71 × 10^-6^) and enolase 1 (ENO1) (*p *= 4.19 × 10^-5^)], while two novel hub genes [RNA binding motif protein 9 (RBM9) (*p *= 1.50 × 10^-4^) and ribosomal protein L30 (RPL30) (*p *= 1.50 × 10^-4^)] may define new central elements in the gene network specific to colon cancer. Gene Ontology (GO) based analysis of the colon cancer-specific gene network and the sub-network that consisted of three-way gene interactions suggested that tumourigenesis in colon cancer resulted from dysfunction in protein biosynthesis and categories associated with ribonucleoprotein complex which are well supported by multiple lines of experimental evidence.

**Conclusion:**

This study demonstrated that IL8, DES and ENO1 act as the central elements in colon cancer susceptibility, and protein biosynthesis and the ribosome-associated function categories largely account for the colon cancer tumuorigenesis. Thus, the newly developed relevancy-based networking approach offers a powerful means to reverse-engineer the disease-specific network, a promising tool for systematic dissection of complex diseases.

## Background

Global gene expression profiling with DNA microarrays has been widely used in deciphering the underlying mechanisms for complex diseases, which have mixed contributions from numerous genetic and environmental factors, and their complex interactions. Now, there are several available approaches that use microarray data to find disease susceptibility genes, based on different metrics that measure the importance of genes involved in pathogenesis. For example, some traditional statistical measures that describe the modelling effects of predictive variables on the studied phenotypes [1], or informatics-based measures that assess the discriminative ability of putative gene features in differentiating phenotypic attributes of samples [2-4]. Recently, we introduced a disease-relevance concept, designed a novel relevance measure, and developed an ensemble decision approach for estimating the strength of (marginal) relevance of a putative gene related to complex diseases [5]. Relevance at large has been well studied in the fields of computer science and decision science. Over the last three decades, increasing interest in applications in a wide range of areas, in particular, machine learning for feature subset selection, has been witnessed. Bell and Wang [6] have reviewed that relevance concepts have evolved considerably, from a simple and intuitive relevance concept for marginally filtering a feature to the sophisticated mathematical formalism of the concept that is quantitative and normalized, and which aims to capture the reality of biological complexities (epistasis or gene-gene interactions). Distinguishing it from the correlation metric commonly used for describing the relationships between genes, the relevance concept can be used to characterize target-dependent behaviour and properties of feature genes, and thus is well suited to identify novel disease-relevance genes and to construct disease-specific gene networks. The former has already been well addressed in the previous report [5], and the latter was the focus of the present study.

Most of the previous efforts to identify molecular determinants of complex diseases have tended not to focus on the intricate interplay between genes responsible for the observed cancer phenotype. Instead, they have mainly used single-gene-based statistical analysis, which is less able to provide a full understanding of the sophisticated interactions between the genetic risk factors. A lesson learned from the increasing evidence coming from model organisms and human studies [7], suggests that interactions among multiple genes/loci contribute broadly to complex traits. Therefore, there is a clear need to develop systematic approaches to unravel the high-order interacting patterns on the high-dimension chips (e.g. microarrays) because they may lead to a better understanding of the complexities involved in diseases.

Gene interaction assay or gene networking have been widely studied [8-11]. The main focus of networking approaches is to build target-independent networks, i.e., directly describing or modelling the pair-wise relationships between genes, without relation to the target (a physiological or disease condition). This includes a variety of approaches, such as Pearson's (or derived) correlation-based approach [12,13], Boolean network [14,15], Bayesian network [16,17], differential equations [11,18] or model free approach [19]. Although these methods have been successfully used to elucidate the functional relationship between genes, they are unlikely to directly output the specific gene networks in response to abnormal physiological conditions such as disease. Recently, several attempts have been made to identify the aberrant behaviour in gene networks in disease conditions. Ergun et al. [20] have applied an approach with two phases to non-recurrent primary and metastatic prostate cancer data. In phase one, a network model of regulatory interactions was reverse engineered. In phase two, the network was used as a filter to determine the genes affected by the condition of interest. The authors identified the androgen receptor (AR) gene among the top genetic mediators, and the AR pathway as a highly enriched pathway for metastatic prostate cancer. Furthermore, they have also demonstrated that the AR gene can be used as a marker to detect the aggressiveness of primary prostate cancers. Daniel et al. [21] have searched for cancer regulatory programs that link transcription factors to target genes that are conditionally activated in specific types or subtypes of cancer. Their results have suggested that alterations in pathways that active some transcription factors might be responsible for the observed gene deregulation and cancer pathogenesis. Segal et al. [22] have developed a module-network approach to identify modules that underlie tumourigenesis. Nevertheless, a comprehensive and systematic approach to constructing *de novo *disease-specific gene networks is lacking, possibly due to no suitable metric to describe disease-driven gene-gene relationships.

The main objective of this study was to evaluate a newly defined disease-driven pairwise relevance metric for identifying interacting gene pairs, followed by constructing disease-specific gene networks related to complex diseases. In some sense, the developed relevance-concept based networking approach was extended from our previously proposed algorithm [5] that aimed to identify disease relevance genes based on a marginal measure or best trees for classification. To describe disease-driven gene-gene relationships, we defined a novel joint relevance measure, called Adjusted Frequency Value (*AFV*) to evaluate the strength of a gene-gene interaction in the gene forest related to complex diseases. We applied the proposed method to analyze a publicly available microarray dataset for colon cancer. First, we constructed a colon cancer-specific gene network. Then, we performed pathway analysis based on curated cell processes, and function enrichment analysis based on Gene Ontology for the gene-gene and three-way gene interactions, in order to establish in which biological processes this network participate, and in which functions associated with colon cancer etiology. Separately, we also identified the hub genes in the constructed gene network for mining the central elements related to colon cancer pathogenesis. Next, a literature searching was carried out to validate the above findings. Finally, the powers of the classifications based on the colon cancer-specific gene network and the colon cancer related gene subset were compared. As a result, we demonstrated that the colon cancer-specific gene network captured the most important genetic interplays in several cellular processes, such as differentiation, mitogenesis, proliferation, apoptosis, inflammation and immunity, which are known to be pivotal for tumourigenesis. Further analysis of the topological architecture of the network identified three known hub cancer genes [interleukin 8 (IL8); desmin (DES) and enolase 1 (ENO1)], while two novel hub genes [RNA binding motif protein 9 (RBM9) and ribosomal protein L30 (RPL30)] may define new central elements in the gene network specific to colon cancer. In addition, Gene Ontology based analysis suggests that the tumorigenesis in colon cancer results from dysfunction in protein biosynthesis and the functional categories associated with ribonucleoprotein complex.

## Results

### Description of the colon cancer data

The proposed method was used to analyze a well-known data set in the microarray literature, colon cancer data, analyzed initially by Alon et al. [23]. It consists of absolute measurements from Affymetrix oligonucleotide arrays, with 62 tissue samples of 2000 human gene expressions (40 tumours and 22 normal tissues).

### Construction of gene forest related to colon cancer

This analysis started with building a gene forest, from which significant gene-gene relationships were extracted. To this end, a 5-fold cross validation resampling strategy was used to construct multiple replicates of training and test sets. In this procedure, colon cancer and normal samples were randomly divided into 5 non-overlapping parts of roughly equal size, denoted as *D*_*i *_(*i *= 1, 2, ..., 5) for colon cancer and *N*_*i *_(*i *= 1, 2, ..., 5) for normal samples, respectively. A combination of *D*_*i *_and *N*_*i *_constituted a test set and the rest of the data were used as the training set. Thus, all combinations produced 25 pairs of training and test sets, {*L*_*d*_, *T*_*d*_} (*d *= 1, 2, ..., 25). By repeating this procedure 20 times, we obtained 500 pairs of data. On each pair, a classification tree was constructed and tested using a computational statistic Matlab toolbox [24], where each gene was a node variable and in this way a gene forest with 500 trees was constructed. We used Gini's diversity index as the criterion for choosing a split. The tree growth was stopped if a further split at the current node did not improve the purity of its child nodes or when there were less than two samples. For the detail of construction of gene forest related to colon cancer, see the Methods section or the previous report [5].

### Distribution of *AFV*s

**Figure 1:**
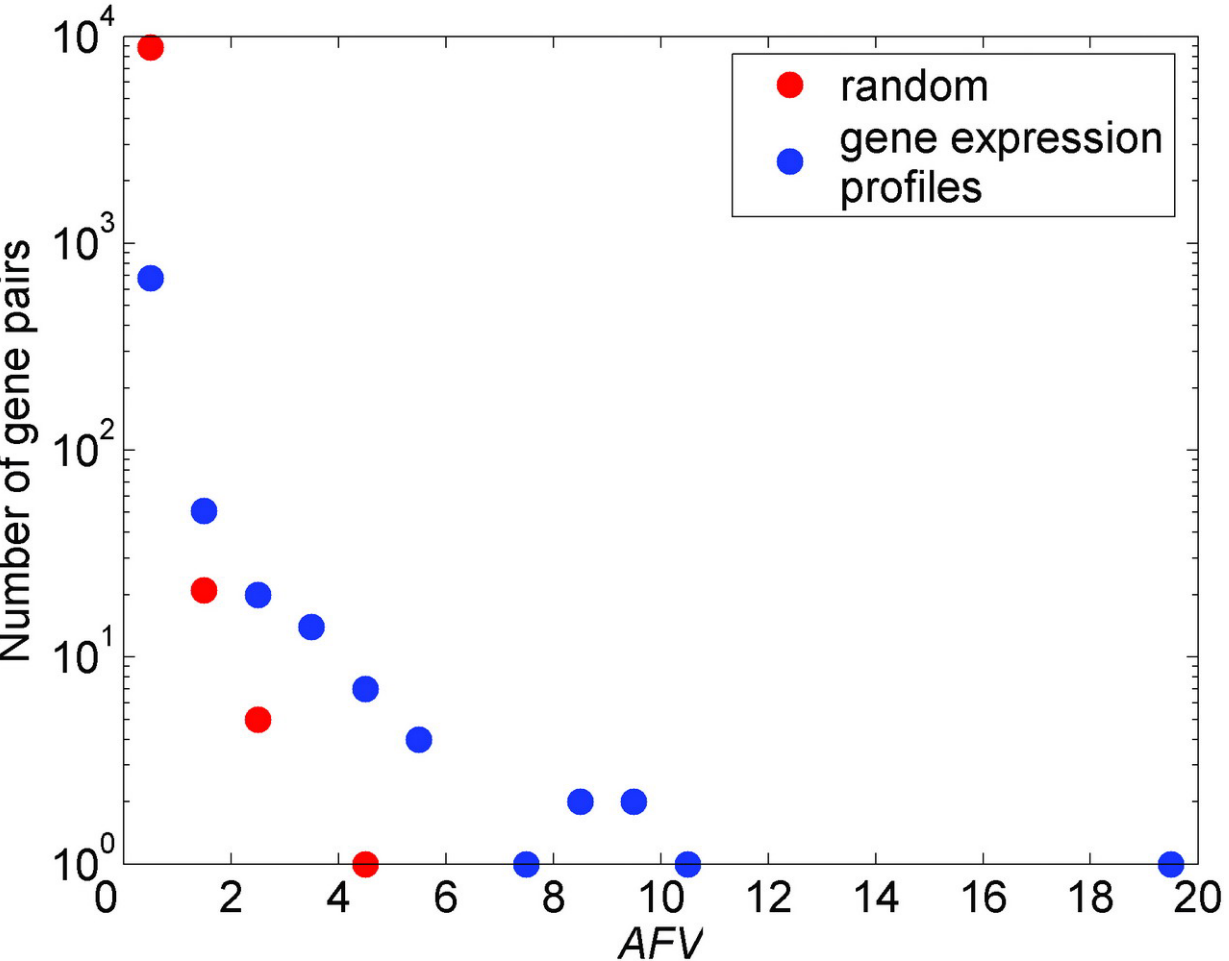
**Distribution of *AFV*s**. Blue circles described the scatter plot of *AFV*s estimated from the field data of 780 gene pairs, while red circles described the scatter plot of *AFV*s estimated from the permutated data of 8881 gene pairs in a random forest of 1000 trees.

From the newly built gene forest, we identified 780 gene pairs (involving 165 genes) appearing in the same trees. Per the definition and formula provided in the Methods section, the *AFV*s for these gene pairs ranged from 0.09 to 19.14, which was generally much smaller than the marginal relevance value that measured the contribution of a single gene feature [5]. The distribution of the 780 gene pairs' *AFV *values is shown with blue circles in Figure 1. In order to determine their statistical significance, we performed 1000 permutations in which the sample labels were randomly shuffled. The estimated empirical null distribution of *AFV *obtained from estimating 8881 gene pairs in 1000 random trees gave the largest value of 4.43 and the threshold for significance level of 0.01 was estimated to be 0.53. The permuted distribution is shown with red circles in Figure 1. Apparently, both curves indicate that this metric follow an extreme value distribution and the curve for the real data shifted to the right of the null distribution. Thus, the gene pairs with *AFV *over the threshold were considered as having significant gene-gene interactions.

### Construction of colon cancer-specific gene network

**Figure 2:**
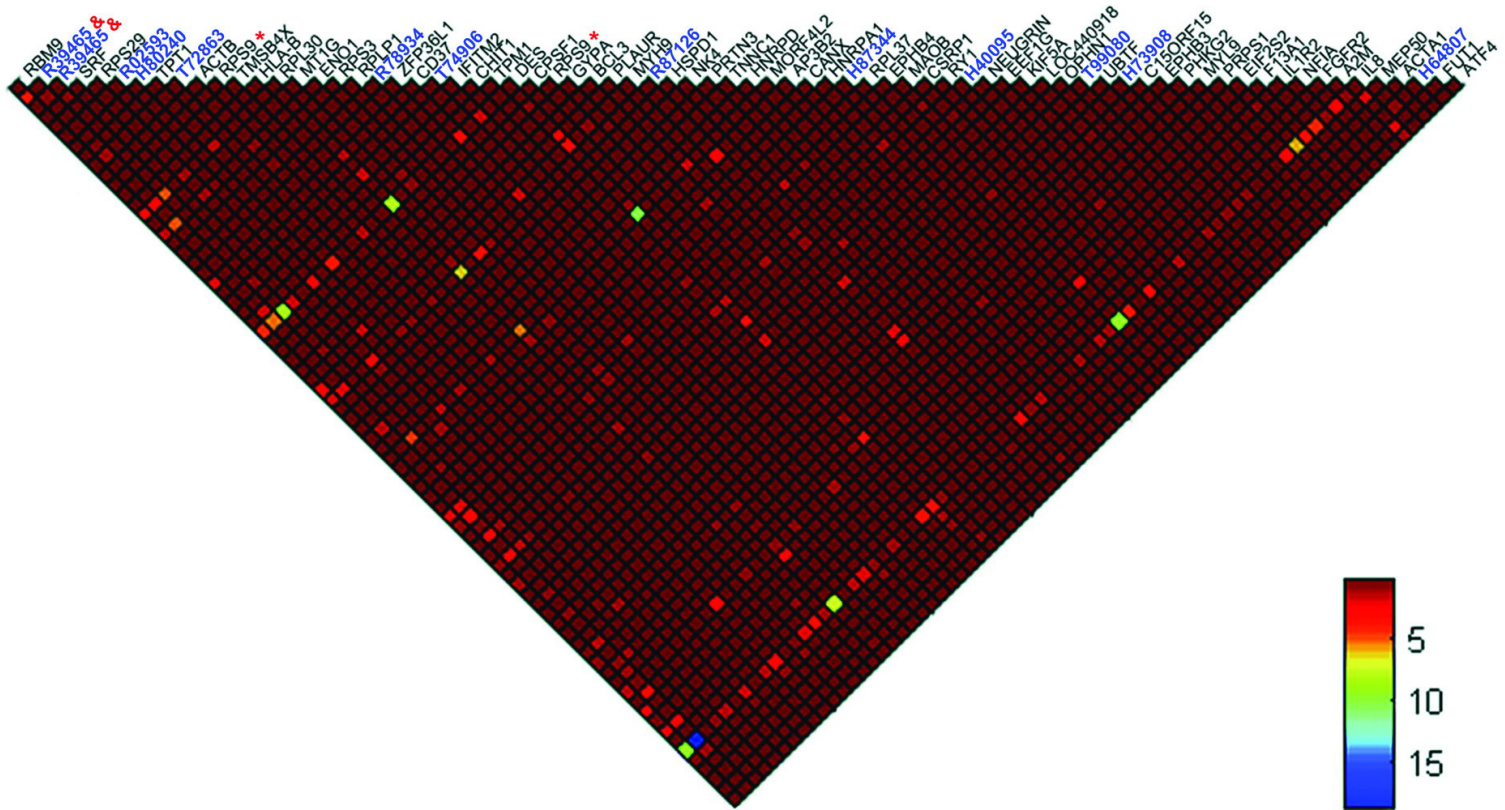
**The heat map for the gene-gene interactions relevant to colon cancer in terms of *AFV***. The interaction strength was depicted by colours, as indicated by the side bar. The gene names or accession numbers (for unknown genes) were shown above the heat map. Symbol '&' indicated the two replicates of a probe, and '*' indicated the two probes correspond to the same gene.

**Figure 3:**
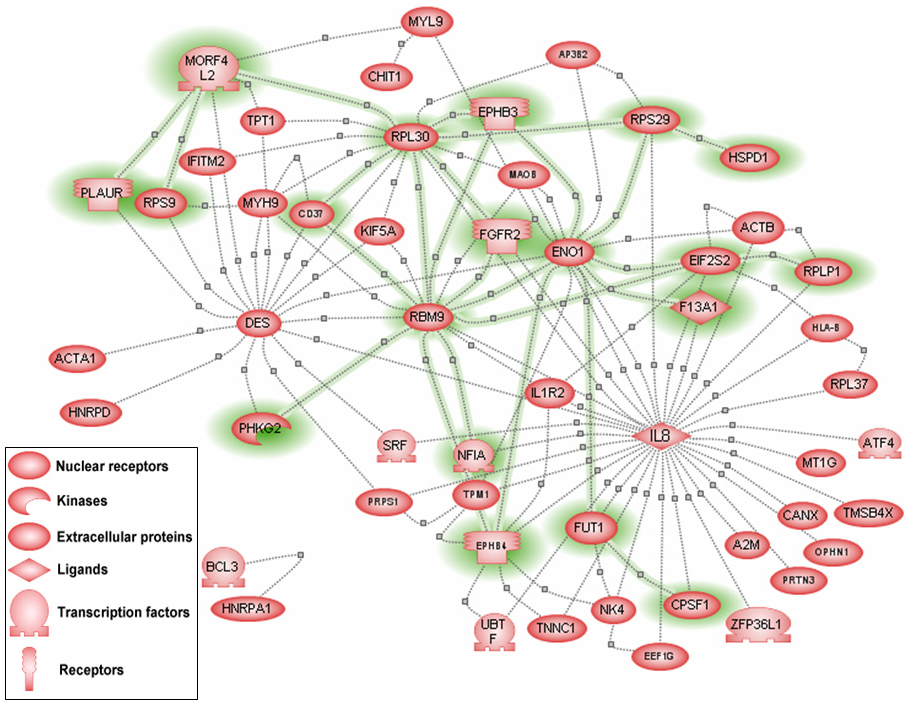
**The colon cancer-specific gene network**. The network was made manually by integrating 109 significant gene-gene interactions among 52 known genes. The functional category "regulation of physiological process" was highlighted with green shadow. Five centres defined by IL8, DES, RPL30, RBM9 and ENO1 were made to be easily visualized. The colour or shape coding of the entities was the same as used in PathwayAssist, as indicated by the bottom bar.

We found 200 significant (*p *≤ 0.01, *AFV *threshold 0.53) colon cancer-specific gene-gene interactions among 74 genes, with the smallest *p *value <1.13 × 10^-4 ^(for details on all the gene pairs, see Additional file 1). All *AFV *values of the 200 significant gene pairs were used to create a graphical representation (Figure 2). The background of the heat map is red, and the *AFV *values are encoded by other colours, as indicated by the side bar. The heat map indicates that only a small proportion (7.40%, 200/2701) reached the significance level, but this number was much higher than expected (0.01 × 2701≈27), which was randomly selected under the null distribution. Intuitively, several genes (e.g. IL8, DES, RPL30, RBM9 and ENO1) had an unusually higher number of significant interacting genes (encoded by non-red colours), which suggests that they may play a central role in the disease process. By annotating the 74 genes to the Entrez Gene [25] and Unigene [26] databases at NCBI, we found 52 known genes that accounted for 109 gene interactions out of the identified 200 gene pairs. To simplify the further bioinformatics analysis, we only focused on the 52 genes whose function had been well characterized and documented in GO. By connecting two genes in each gene pair, we constructed an un-weighted gene network for colon cancer (Figure 3). One can easily identify that five genes (IL8, DES, RPL30, RBM9 and ENO1) had the highest connectivity scores. IL8, a chemotactic and inflammatory cytokine (a ligand), had 33 connections with the 52 known genes; next was DES, a type III intermediate filament found near the Z line in sarcomeres, which had 17 connections.

The functional implications of the constructed network remained to be elucidated. Thus, we used 'Functional Annotation' in DAVID Bioinformatics Resources to perform functional enrichment analysis based on Gene Ontology [27]. We defined the 74 genes as the test set and the entire 2000 genes as the background. We set a minimal node size of five genes from the test set, and a nominal significance level of 0.05, given by the EASE Score method, a modified Fisher Exact test. We identified 13 significant GO terms, as shown in Table 1. In order to identify more specific functions, we eliminated the redundant but broad terms among the 13 GO terms. Finally, we obtained seven more specific GO terms (shown in bold type in Table 1). From the two dimensions 'Cellular Component' and 'Molecular Function', we found that the pathogenesis of colon cancer was consistently linked to ribosome (associated categories such as 'ribosome', 'ribonucleoprotein complex' and 'structural constituent of ribosome'). Based on the dimension 'Biological Process', we concluded that 'protein biosynthesis' largely accounted for colon cancer tumourigenesis. These conclusions are well supported by multiple lines of experimental evidence. One study has demonstrated that there is increased synthesis of ribosomes in colorectal tumours, and that this increase is an early event in colon neoplasia [28]. In another recent study [29], it has been shown that perturbation of specific ribosomal proteins is likely to promote certain genetic diseases and tumuorigenesis.

**Table 1:** The GO terms that significantly enriched with gene-gene interactions. In the bold style are the more specific GO terms

Category	GO term	*p*	Description
Biological Process	GO:0009059	0.0006	macromolecule biosynthesis
	**GO:0006412**	**0.0066**	**protein biosynthesis**
	GO:0044249	0.0071	cellular biosynthesis
	GO:0009058	0.0143	biosynthesis
	**GO:0006936**	**0.0245**	**muscle contraction**
	**GO:0016043**	**0.0306**	**cell organization and biogenesis**
Cellular Component	GO:0043228	0.0007	non-membrane-bound organelle
	GO:0043232	0.0007	intracellular non-membrane-bound organelle
	**GO:0005840**	**0.0022**	**ribosome**
	**GO:0030529**	**0.0099**	**ribonucleoprotein complex**
	**GO:0043234**	**0.0151**	**protein complex**
Molecular Function	GO:0005198	0.0032	structural molecule activity
	**GO:0003735**	**0.0055**	**structural constituent of ribosome**

### Identification of hub colon cancer genes

We used a Poisson distribution to identify statistically significant hub nodes in the colon cancer specific network. Under the null hypothesis that the 52 genes were randomly connected, a gene with >10 connections in a random network was considered a rare event with probability of 0.0046. Thus, we set this threshold to claim a hub gene. By this criterion, we identified five hub genes: IL8 (33 connections; *p *≈ 0), DES (17 connections; *p *= 2.71 × 10^-6^), RBM9 (15 connections; *p *= 4.19 × 10^-5^), RPL30 (14 connections; *p *= 1.50 × 10^-4^) and ENO1 (14 connections; *p *= 1.50 × 10^-4^). Even after adjusting for the number of genes tested, the five genes remained to be valid hub genes with highly significant connectivity. Their corresponding Bonferroni-corrected *p *values were ≈0, 1.41 × 10^-4^, 2.18 × 10^-3^, 7.79 × 10^-3^, 7.79 × 10^-3^, respectively. Three of the five hub genes (IL8, DES and ENO1) are proved cancer-related hub genes, while knowledge for the remaining two genes waits to be expanded. The detailed cross-talks with the three proved cancer-related hub genes are listed in Table 2.

**Table 2:** The gene interactions that involved 3 known cancer genes in colon cancer-specific gene network

Hub gene	Gene	*AFV*	*p*	Hub gene	Gene	*AFV*	*p*
IL8	EPHB4	9.53	0.0001	DES	MORF4L2	10.23	0.0001
	RBM9	9.46	0.0001		RPL30	8.37	0.0001
	ENO1	7.54	0.0001		RBM9	4.38	0.0002
	EIF2S2	5.88	0.0001		TPT1	4.03	0.0002
	IL1R2	4.34	0.0002		PRPS1	3.69	0.0002
	MAOB	3.98	0.0002		IL8	3.69	0.0002
	F13A1	3.72	0.0002		CD37	3.59	0.0002
	DES	3.69	0.0002		MYH9	3.53	0.0002
	TPM1	3.64	0.0002		PLAUR	2.17	0.0007
	RPL30	3.45	0.0002		KIF5A	1.71	0.0008
	NK4	3.41	0.0002		SRF	1.58	0.0011
	PRPS1	2.88	0.0002		IFITM2	1.53	0.0011
	ACTB	2.59	0.0003		RPS9:15*	1.22	0.0023
	FGFR2	2.52	0.0003		RPS9:275*	0.87	0.0036
	HLA-B	2.06	0.0007		ENO1	0.83	0.0039
	FUT1	1.92	0.0008		HNRPD	0.83	0.0039
	TNNC1	1.35	0.0016		PHKG2	0.82	0.0041
	RPS29	1.33	0.0018		ACTA1	0.70	0.0051
	RPLP1	1.29	0.0019				
	EEF1G	1.24	0.0023	ENO1	IL8	7.54	0.0001
	RPL37	1.15	0.0024		F13A1	2.69	0.0003
	CANX	1.07	0.0026		RBM9	1.21	0.0023
	OPHN1	1.06	0.0026		RPS29	1.00	0.0032
	ATF4	0.97	0.0033		DES	0.83	0.0039
	MT1G	0.93	0.0036		AP3B2	0.79	0.0045
	A2M	0.86	0.0037		EPHB4	0.76	0.0046
	NFIA	0.84	0.0037		MAOB	0.72	0.0048
	ZFP36L1	0.73	0.0047		TPM1	0.72	0.0050
	SRF	0.72	0.0048		EPHB3	0.65	0.0064
	PRTN3	0.71	0.0050		ACTB	0.61	0.0074
	UBTF	0.67	0.0060		EIF2S2	0.61	0.0074
	CPSF1	0.65	0.0064		MYL9	0.58	0.0081
	TMSB4X	0.54	0.0098		FUT1	0.56	0.0090

*the two probes correspond to the same gene RPS9

The protein encoded by Interleukin 8 (IL8), is a member of the CXC chemokine family. This chemokine is one of the major mediators of the inflammatory response. IL8 can promote cell proliferation and migration through metalloproteinase-cleavage proHB-EGF in human colon carcinoma cells [30], and induction of IL8 preserves the angiogenic response in HIF-1alpha-deficient colon cancer cells [31]. Desmin (DES) encodes a muscle-specific class III intermediate filament. Mutations in this gene are associated with desmin-related myopathy, a familial cardiac and skeletal myopathy (CSM), and with distal myopathies. It is also a negative marker for colon cancer discrimination [32]. Enolase 1, more commonly known as alpha-enolase, is a glycolytic enzyme expressed in most tissues. It is a homodimer composed of 2 alpha subunits. Its gene, the ENO1, also encodes the Myc-binding protein-1, which downregulates the activity of c-myc protooncogene [33]. However, there are few studies that can establish the hub roles of the remaining two genes (RPL30 and RBM9). The ribosomal protein L30 (RPL30) encodes a ribosomal protein that is a component of the 60S subunit. Disease specific humoral immune responses against TBP-1, p27(BBP), and RPL30 have been induced in patients with hepatocellular carcinoma (HCC), and the antibodies against these antigens may be also used as tumour markers [34]. Gene RBM9 encodes an RNA binding protein that is thought to be a key regulator of alternative exon splicing in the nervous system and other cell types [35]. The protein also interacts with the estrogen receptor 1 transcription factor and regulates estrogen receptor 1 transcriptional activity [35]. However, there is a dearth of information that show its direct effects on the tumourigenesis in cancer.

### Pathway analysis of hub colon cancer genes

**Figure 4:**
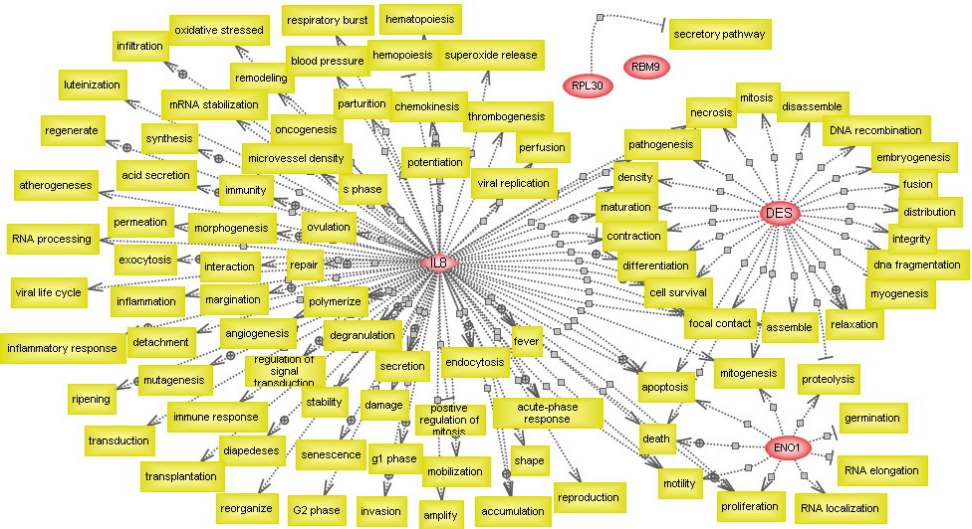
**The knowledge-based gene network involving all cellular processes directly linked to the hub genes**. This network was constructed by finding out all cellular processes directly linked to the 5 hub colon cancer genes using PathwayAssist software.

To validate the newly identified five hub genes, we performed a pathway analysis using PathwayAssist software (Stratagene, La Jolla, CA, USA) [36]. The knowledge-based gene network (Figure 4) was constructed by finding out all cellular processes directly linked to the hub genes. Based on this analysis, IL8, DES and ENO1 are proven central elements in this network, with 92, 24 and nine links, respectively. However, there are insufficient data to prove the hub roles of RPL30 (one link) and RBM9 (no link), as revealed by the above *AFV*-based networking, and these two genes may define new central elements in the gene network specific to colon cancer. Based on the cellular processes to which the hub genes were linked, the colon cancer-specific gene network captured the most important genetic interplays in several cellular processes such as differentiation, mitogenesis, proliferation, apoptosis, inflammation and immunity, which are known to be pivotal for tumourigenesis.

**Figure 5:**
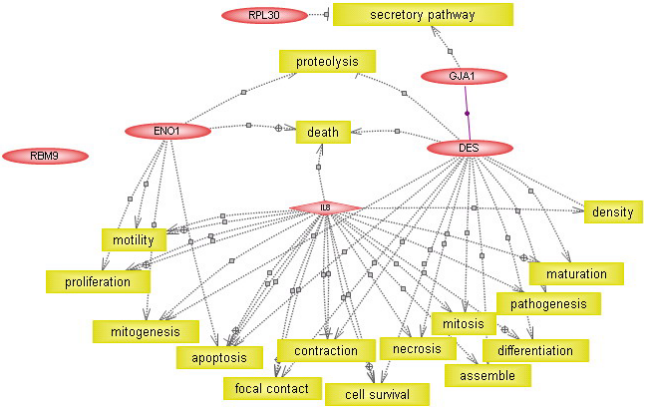
**The knowledge-based gene network involving all cellular processes (or proteins) that link the hub genes**. This network was constructed by finding all the cellular processes shared by the hub genes by implementing the option "Find all shortest paths between selected entities" in PathwayAssist software.

We also conducted a pathway analysis to identify all cellular processes (or proteins) that link the five hub genes by implementing "Find all shortest paths between selected entities" in PathwayAssist Software. Again, IL8, DES and ENO1 were the central elements (Figure 5). Interestingly, in this network, RPL30 and DES can be linked through GJA1 (connexin-43), the major protein of myocardial gap junctions, which are thought to have a crucial role in the synchronized contraction of the heart and in embryonic development. It was also interesting to note that the common cellular processes for the three hub genes IL8, DES and ENO1 greatly varied from cell proliferation and differentiation to maturity and death. This may have been due to the large number of cellular functions to which IL8 was linked (see also Figure 4).

### High-order interactions in the colon cancer-specific gene network

In the colon cancer-specific gene network, 76 three-way interactions (triangles) among 60 genes were identified by an exhaustive searching algorithm for the network motifs. Based on 1000 random networks, only the triangle structure, which included all possible edges between the three nodes, was over-represented (*p* = 0.012) in this network at the significance level of 0.05 using MAVisto software [37]. Hence, we focused on the triangle as the structural element in further analysis. In addition, we also searched for larger *n*-cliques, which were complete sub-graphs with *n *nodes. A maximum-size 5-clique was found that described the dense cross-talks between five genes: CD37, DES, MYH9, RBM9 and RPL30. However, this 5-clique could not be fully confirmed by our current knowledge of the five molecules, and further experimental validation is required.

Then, an enrichment analysis based on GO was performed. We defined the functional facets of the 60 genes using the DAVID resources [27], and the parameters were set as described above. We identified 11 GO functional categories, of which the terms, 'ribosome', 'ribonucleoprotein complex', 'structural constituent of ribosome' and 'protein biosynthesis', were the most specific functionalities, as shown in Table 3. These results were consistent with the enrichment analysis of two-way interactions, which suggested that the above categories largely captured the functional facets of the colon cancer specific gene network.

**Table 3:** The GO terms that significantly enriched with three-way interactions. In the bold style are the more specific GO terms

Category	GO term	*p*	Description
Biological Process	GO:0009059	0.0014	macromolecule biosynthesis
	**GO:0006412**	**0.0056**	**protein biosynthesis**
	GO:0044249	0.0080	cellular biosynthesis
	GO:0009058	0.0150	biosynthesis
Cellular Component	GO:0043228	0.0018	non-membrane-bound organelle
	GO:0043232	0.0018	intracellular non-membrane-bound organelle
	**GO:0005840**	**0.0039**	**ribosome**
	**GO:0043234**	**0.0077**	**protein complex**
	**GO:0030529**	**0.0301**	**ribonucleoprotein complex**
Molecular Function	GO:0005198	0.0074	structural molecule activity
	**GO:0003735**	**0.0102**	**structural constituent of ribosome**

### Comparison of classification performances

**Figure 6:**
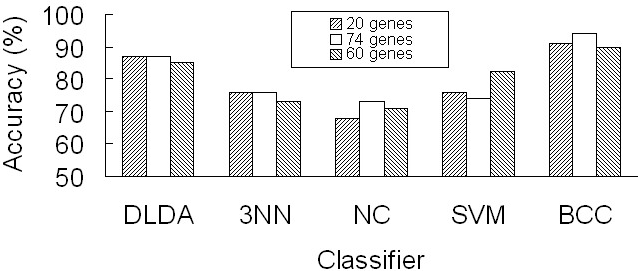
**Comparison of the mean classification performances of the three gene pools**. The 20 highly significant colon cancer relevant genes were identified in our previous study. The 74 and 60 genes were extracted from the gene network based on gene-gene interactions and three-way interactions, respectively.

In our previous study [5], we identified 20 highly significant colon cancer relevant genes based on a marginal relevance index that measured their separate contribution to the gene forest for classification. Logically, the gene networks that included both the marginal and joint contributions of the colon cancer genes may better define the susceptibility risk for developing colon cancer. To verify this hypothesis, we compared the three gene sets: the 20 genes that extracted from our previous study, the 74 genes that extracted from gene-gene interactions and 60 genes that extracted from three-way interactions. We estimated the average accuracy of the three sets by leave-one-out validation using 5 classifiers: diagonal linear discriminate analysis (DLDA), 3 nearest neighbours (3NN), nearest centroid (NC), support vector machine (SVM) and Bayesian compound covariate (BCC), which were all implemented using the BRB-Arraytools software version 3.5.0 stable release [38]. As a result, although the differences were not statistically significant, the gene network with gene-gene interactions, in most of the classifiers, had an equal or better power than the 20 marginally relevant genes in classifying tissue samples, or the gene set defined by three-way interactions, as conceptually this set was a subset of the data defined by two-way interactions (Figure 6). This result suggested that gene network may contain additional contributions from the gene-gene interactions and the three-way interactions.

## Discussion

Most cancers, including colon cancer, are complex disorders that can be caused by multiple genes and their complex interactions. With the advance of high throughput technologies, it is now feasible to reversely engineer the underlying genetic networks that describe the complex interplay of molecular elements that lead to complex diseases. In this study, we proposed and evaluated a novel relevance-concept metric (*AFV*) for identifying joint contributions to complex diseases based on genome-wide gene expression profiles, followed by constructing disease-specific gene networks. This approach was partly an extension of our previously proposed algorithm [5], which aimed to identify disease relevant genes based on a marginal measure or best trees for classification. In order to establish the power of the novel pair-wise relevance metric (*AFV*), we analyzed genome-wide colon cancer microarray data. Most of the results were supported by previous findings, and some interesting results can be considered as hypothesises, which require further experimental validation.

Currently, two innovative concepts, disease relevance and system biology, and the corresponding computational algorithms are intriguing and appealing to map the complexities in complex disease and are deemed to offer new promises for promoting deep dissection of complex disease in the new century. The concept of disease relevance, first proposed and defined by us [5], was derived from a similar concept widely used in a range of areas, in particular, in machine learning of industrial systems and social-economic systems. This concept tactically exploits the universal axiom of "a whole is larger than the sum of its integral components" for explaining the genetic complexities of biological systems. The purposes of introducing the relevance concept into the proposed approach for disease-specific gene networking are: (i) to characterize the target-dependent behaviour and properties of gene-gene interactions that are largely ignored in the prevalent correlation metric; and (ii) to define a statistic that measures the degree of pair-wise relevance of a gene pair for reversely reconstructing genetic networks for complex disease. The second concept, system biology, is a fashionable label for a new generation of large-scale experiments (e.g. the genome-wide transcriptional profiling used in this study) [39], which study biological systems by holistically viewing the structure of the system and its response to individual perturbations [40]. These perceptions are conceptually intriguing because they provide ways of better understanding complex disease [5] and are thus applauded in the fields of computational biology [40-43] and applied domains (e.g. cancers [44], atherosclerosis [45] and drug discovery [9,10]).

To our knowledge, this study is a pioneering attempt at developing a relevance concept based systematic approach to reversely engineer the underlying genetic networks that describe the complex interplay of molecular elements that lead to complex diseases. The main advantages of the proposed method are as follows: (i) Current networking approaches mainly focus on building genetic networks at large without probing the interaction mechanisms specific to a physiological or disease condition. However, our approach can identify the joint contribution of two genes to complex diseases and construct complex disease-specific gene networks. (ii) The novel relevant metric *AFV *was not the directly calculated correlation between two individual genes, but was drawn from the same gene subsets (or pathways) that had high discrimination between different phenotypes. In this study, there were 2000 gene-expression patterns. If we used correlation-based methods, there would be 1,999,000 possible interactions. However, there were only 780 gene pairs extracted from our constructed gene forest. Furthermore, a correlation metric is commonly used for describing the relationships between genes, whereas the relevance concept can be used to characterize the target-dependent behaviour and properties of a feature gene, and thus is well suited to identify novel disease-relevant genes and to construct disease-specific gene networks. (iii) During tree-building we did not perform either pre- or post-pruning in order to minimize the risk of losing any important feature gene because of the limited sample sizes. Thus, we identified most, if not all genes related to colon cancer (including trivial genes), even if some genes might be removed from the ensemble decision analysis. (iv) The proposed method can be straightforwardly applied to different types of data of high dimension in nature. For example, in a recent study [46], we applied the similar tree-based ensemble method for mapping multiple loci for rheumatoid arthritis (RA) via analysis of 746 multiplex families genotyped with >5000 genome-wide single nucleotide polymorphisms (SNPs). We successfully identified 41 significant SNPs relevant to RA, 25 associated genes and a number of important SNP-SNP interactions (SNP patterns). Many findings (loci, genes and interactions) have experimental support from previous studies while novel findings may define unknown genetic pathways for this complex disease.

To further investigate the efficiency of our approach, we also analyzed other independent microarray data for prostate cancer. The identified genes and biological processes were highly related to prostate cancer, which was supported by multiple lines of experimental evidence. The detailed results are given in Additional file 2. Thus, both a recent study [46] and the present study demonstrated that the proposed pair-wise relevance metric was useful when applied to analysis of genome-wide data and offered a promising measure to reversely engineer the underlying genetic networks for complex human diseases.

## Conclusion

It can be seen that most of the previous efforts for identifying molecular determinants for complex diseases less often focused on the intricate interplays of genes responsible for the observed cancer phenotype, but were largely implemented using single-gene based statistical analysis approaches that are less efficient in providing a deep understanding of the sophisticated interplays between these genetic risk factors. In this study, we proposed and evaluated a novel relevance-concept metric (*AFV*) to assess the joint contributions of genes for complex diseases, followed by constructing disease-specific gene networks related to complex diseases. After that, we identified the hub genes of the constructed gene network, and then performed functional annotation and literature searching to investigate the relationship of the local elements with the studied disease. Next, we mined the three-way gene interactions (motifs), and then conducted function enrichment analysis of gene-gene and three-way gene interactions to find out the global characteristics related to disease pathogenesis. Application to a colon cancer microarray dataset demonstrated that the colon cancer-specific gene network captured the most important genetic interplays in several cellular processes such as differentiation, mitogenesis, proliferation, apoptosis, inflammation and immunity that are known to be pivotal for tumorigenesis. Further analysis of the topological architectures of the network identified three known hub cancer genes (IL8; DES and ENO1), while two novel hub genes (RBM9 and RPL30) may define new central elements in the gene network specific to colon cancer. Gene Ontology based analysis of the colon cancer-specific gene network and the subnetwork consisted of three-way gene interactions suggested that the tumorigenesis in colon cancer was resulted from dysfunction in 'protein biosynthesis' and the categories associated with ribonucleoprotein complex. In conclusion, this study demonstrated that the newly developed relevancy-based networking approach offered a powerful means to mine joint contributions of genes for complex diseases and reverse-engineered the de nova disease-specific network, a promising tool for systematic dissection of complex diseases.

## Methods

### Definitions

A gene chip, a snapshot of the mRNA transcriptional activities of *p *genes in *n *tissue samples collected from either cancer or health patients, mathematically can be described by a *n *× *p *matrix, *X *= (*x*_*ij*_), where *x*_*ij *_represents the expression level for the *j*th gene (*g*_*j*_) on the *i*th sample (*X*_*i*_). The data for each sample consists of a vector of expression profile, *X*_*i *_= (*x*_*i*1_, *x*_*i*2_, ..., *x*_*ip*_) and a category label (*y*_*i*_) describing the physiological (or pathological) condition that a subject has (e.g. diseased or healthy).

In our previous studies, we developed a systematic ensemble decision approach for hunting for disease genes using microarray expression profiling. The basic strategies were as follows. (i) To build all possible gene subsets by repeated learning and testing of multiple resampling-generated training and test datasets that were used for mapping the underlying molecular pathways that lead to complex disease. As a disease relevant gene subset was obtained by using a tree-based recursive partitioner, we named gene forest for the pool of such gene subsets; (ii) To identify all the disease relevant genes based on the behaviour and role of the molecular features in the gene forest. To this end, we defined a marginal relevance index that measured its contribution to the gene forest and derived a formula called ensemble vote, *FV*, which was the weighted frequency estimate of a putative disease gene that appeared in the trees of the forest. In the present study, we extended the ensemble-decision approach to identify disease-relevance gene-gene interactions and to build disease-specific gene networks.

#### Definition 1

The relevance of a gene-gene interaction pair (*g*_*i*_, *g*_*j*_) for a disease is defined as their joint contribution to the gene forest for the disease. We claim that the gene-gene interaction is relevant to the disease if *g*_*i *_and *g*_*j *_appear simultaneously at a significantly higher frequency in the same trees in the forest than that in a random forest that corresponds to the null hypothesis of no gene-disease relevance.

#### Definition 2

Given an undirected graph, *G*, which comprises a set of vertices (genes) *V *and a set of edges, *E *⊆ *V *× *V*, the graph, *G*, is a disease-relevant gene network if every edge <*ν*_1_, *ν*_2_> in *E *is a disease-relevant gene-gene interaction.

### Construction of gene forest related to disease

First, a resampling technique was employed to build up pairs of training and test sets, {*L*_*d*_, *T*_*d*_} (*d *= 1, 2, ..., *m*), for learning and testing, respectively. Then, a binary decision tree was grown on *L*_*d *_by a recursive partition algorithm. At each non-leaf node, a decision was made with regard to the choice of a feature gene and a threshold value (cut-off) such that the class impurity was reduced to a minimum when a branch was made by an induction rule. After the optimal bifurcation was made, the microarray samples were divided into two non-overlapping subsets (two child nodes). The same process was conducted successively until the stopping criteria for tree growth were satisfied. For each tree grown, it was tested on the holdout set *T*_*d *_to evaluate its discriminating power for classification. This process was repeated on each pair of {*L*_*d*_, *T*_*d*_} (*d *= 1, 2, ..., *m*), which consequently resulted in a decision forest with *m *trees. In each tree, all the genes for bifurcation at non-leaf nodes composed a disease relevant gene subset (pathway), which denoted as *G*_*d *_(*d *= 1, 2, ..., *m*). All *G*_*d *_extracted from the *m *trees composed the gene forest. The aim of this step was to identify most, if not all genetic pathways that lead to complex disease.

### Construction of disease-specific gene network

Based on the gene forest established in the previous step, we extracted all gene pairs in the same gene subsets. In order to quantify the joint contribution of a gene pair, according to **Definition 1 **we designed a novel pair-wise relevancy metric, called Adjusted Frequency Value (*AFV*), which was formulated as follows:

$$AFV\left(g_{i},g_{j}\right)=100\times \frac{\sum_{d}\omega_{d}I\left(g_{i},g_{j}|G_{d}\right)}{\sum_{d}\omega_{d}},$$

where *I*(*g*_*i*_, *g*_*j*_|*G*_*d*_) was an indicator function and *G*_*d *_was the gene subset that contains the gene pair:

$$I\left(g_{i},g_{j}|G_{d}\right)=\{\begin{array}{ll} 1, & \text{if both }g_{i}\text{ and }g_{j}\text{ appear in the }d\text{th tree }(G_{d})\\ 0, & \text{otherwise}. \end{array}.$$

A weight, *ω*_*d*_, was a measure for the classification performance of *G*_*d *_on a test set, such as the accuracy rate used in this study. In short, one gene pair's *AFV *value was weighted frequency of the two genes appear simultaneously in the same trees in the forest.

Because the asymptotic distribution of *AFV *could not be derived analytically, we resorted to a permutation approach to obtain its empirical null distribution. In the permutation approach, we randomly assigned a label (phenotype), *y*_*i*_, to each microarray and then the same procedures for the field data were applied to the permutated data. Given the empirical *AFV*s and a user-specified significance level (e.g. *α *= 0.05 or 0.01), a critical value for *AFV *was determined by its (1-*α*)% percentile in the simulated null distribution. A gene-gene interaction was disease relevant if it's $AFV\geq AFV_{\alpha }^{0}$, the threshold value at significance level *α *(one-tailed). According to **Definition 2**, if a gene network was built in such ways that every presented edge was a disease-relevant gene-gene interaction, it was a gene network specific to the disease, a sub-network enriched with pathogenic pathways that lead to the disease.

In order to characterize the functional facets of the constructed disease-relevant gene network, we performed functional enrichment analysis based on GO using 'Functional Annotation' in DAVID Bioinformatics Resources [27]. All the 2000 genes analyzed in this study were used as the background. The probability of a GO term enriched with the gene-gene interactions was assessed by the EASE Score method, a modified Fisher Exact test. A smaller EASE Score was related to a higher likelihood of enrichment of a GO term with the gene-gene interactions. In this study, to avoid the possible loss of the true positive results, we did not perform multiple-test correction for the multiple GO terms evaluated. Therefore, the *p*-value quoted should be considered as a heuristic measure, useful for roughly rating the relative enrichment of each GO term. We removed all redundant terms if all the genes annotated to a term were also annotated to a child term. In this case, we retained the child term because its function was more specifically defined.

### Identification of hub disease genes

The topology and properties for most cellular networks were largely determined by a relatively small number of hub nodes (genes), which, in the context of a disease-relevant network, were key genes that lead to disease or maintaining health physiological condition. Connectivity (the number of links) was often used to measure importance of a hub node, which, in random network, follows a Poisson distribution [47]. We used the following formula to determine whether a node could be categorized as a hub node. Suppose that *p*_1 _was the probability of connecting any two nodes in a random network with *n *nodes, the probability of connectivity of equal or larger than *t *was as follows:

$$p\left(x\geq t\right)=1-p\left(x<t\right)=1-\sum_{k=0}^{t-1}\frac{\lambda^{k}e^{-\lambda }}{k!},$$

where *λ *= *n *× *p*_1_; *p*_1 _was estimated using the number of links in the constructed disease-specific gene network divided by the number of all possible links. We claimed a hub gene if its *p *value was smaller than the nominal significance *α*.

### Pathway analysis of hub colon cancer genes

To identify more specific pathways associated with the underlying pathogenic mechanisms of colon cancer, we used PathwayAssist software (Stratagene, La Jolla, CA, USA) to find all the cellular processes linked to the hub colon cancer genes using the option "Find all entities connected to selected entities (Expand Pathway)". Then, we identified all the cellular processes shared by the hub genes by implementing the option "Find all shortest paths between selected entities".

### High-order interactions in the colon cancer-specific gene network

We further investigated high-order gene interactions. In this study, triangles (three-way interactions), which have all possible edges among the three vertices, were extracted from the network by an exhaustive searching algorithm and tested using MAVisto software [37]. Then, in order to characterize the functions of these triangles, we annotated the gene pool of the triangles to GO, and assessed the enrichment of each GO term with these genes, using the DAVID resources [27], as described above. Again, for the reasons specified above, we did not perform multiple tests for multiple GO terms evaluated.

**Figure 7:**
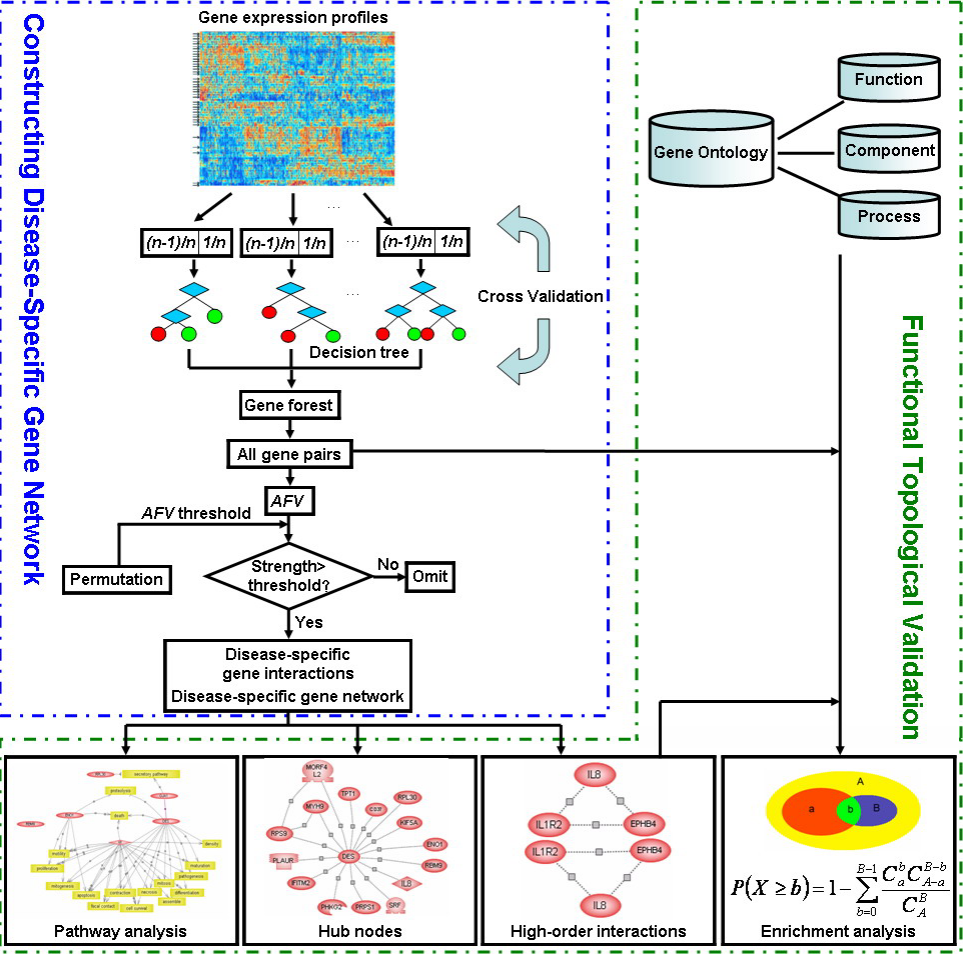
The algorithm flow chart of the proposed network approach.

In order to better explain the novel network approach, we also made a graphic algorithm flow chart, as shown in Figure 7.

## Authors' contributions

This study was undertaken by a collaborative team of several institutes as indicated. WJ, XL, SR and BY conceived of the proposal of the study, conducted the study and drafted the manuscript. The remaining authors participated in writing the computing codes and applied the data mining strategy to the field datasets. All authors participated in reading, approving and revising the manuscript.

## Supplementary Material

Additional file 1 The *AFV *values of the 200 colon cancer-specific gene interactions. Click here for file

Additional file 2 Application of the novel network approach to prostate cancer microarray data. Click here for file
