# Supplementary material for: Constructing disease-specific gene networks using pair-wise relevance metric: Application to colon cancer identifies interleukin 8, desmin and enolase 1 as the central elements
Source: BMC Syst Biol. 2008 Aug 10;2:72. doi: 10.1186/1752-0509-2-72 (PMC2535780; doi:10.1186/1752-0509-2-72)
Supplement: Additional file 1 — The AFV values of the 200 colon cancer-specific gene interactions. [file 1752-0509-2-72-S1.pdf]

Table 1. The *AFV* values of the 200 colon cancer-specific gene interactions

Gene (Symbol or Accession number)	Gene (Symbol or Accession number)	*AFV*	*p*
R39465	IL8	19.144	0.0001
**DES**	**MORF4L2**	10.233	0.0001
**EPHB4**	**IL8**	9.529	0.0001
**RBM9**	**IL8**	9.4601	0.0001
R39465	DES	8.4874	0.0001
**RPL30**	**DES**	8.3669	0.0001
**ENO1**	**IL8**	7.544	0.0001
RPL30	R87126	5.9911	0.0001
**EIF2S2**	**IL8**	5.8792	0.0001
**RPL30**	**MORF4L2**	5.2267	0.0001
R39465	DES	5.225	0.0001
R39465	ENO1	4.9874	0.0001
R39465	RPL30	4.8273	0.0001
R39465	MORF4L2	4.5364	0.0001
**RBM9**	**DES**	4.3832	0.0002
**IL1R2**	**IL8**	4.3401	0.0002
**TPT1**	**DES**	4.032	0.0002
RBM9	R39465	4.0199	0.0002
**MAOB**	**IL8**	3.9786	0.0002
**F13A1**	**IL8**	3.7152	0.0002
**DES**	**PRPS1**	3.6945	0.0002
**DES**	**IL8**	3.6928	0.0002
**TPM1**	**IL8**	3.6394	0.0002
ENO1	R87126	3.5947	0.0002
**CD37**	**DES**	3.5878	0.0002
RY1	IL8	3.5482	0.0002
**DES**	**MYH9**	3.5344	0.0002
**RPL30**	**IL8**	3.4501	0.0002
**NK4**	**IL8**	3.4053	0.0002
R39465	F13A1	3.3072	0.0002
DES	NEUGRIN	3.2934	0.0002
**EPHB4**	**IL1R2**	3.0352	0.0002
R39465	MAOB	2.987	0.0002
**PRPS1**	**IL8**	2.875	0.0002
**RBM9**	**MYH9**	2.789	0.0002
RPS29	R87126	2.7356	0.0003
**ENO1**	**F13A1**	2.6857	0.0003
R39465	RY1	2.6753	0.0003
R39465	RPL30	2.6375	0.0003
R39465	NEUGRIN	2.6134	0.0003
**ACTB**	**IL8**	2.591	0.0003
**RBM9**	**RPL30**	2.572	0.0003
**FGFR2**	**IL8**	2.5204	0.0003
**ACTB**	**EIF2S2**	2.4119	0.0003
R87126	AP3B2	2.4016	0.0005
R39465	FGFR2	2.2966	0.0005
**DES**	**PLAUR**	2.1726	0.0007
**RBM9**	**IL1R2**	2.1589	0.0007
R02593	DES	2.1554	0.0007
R87126	EPHB3	2.1038	0.0007
R87126	C15orf15	2.0797	0.0007
**HLA-B**	**IL8**	2.0642	0.0007
**RBM9**	**EPHB4**	1.9729	0.0008

**IL8**	**FUT1**	1.923	0.0008
R78934	IL8	1.9041	0.0008
R39465	EPHB4	1.8404	0.0008
IL8	MEP50	1.8318	0.0008
R39465	R87126	1.83	0.0008
**RBM9**	**FGFR2**	1.818	0.0008
**RBM9**	**CD37**	1.8128	0.0008
**CD37**	**MYH9**	1.7457	0.0008
R02593	PLAUR	1.725	0.0008
**DES**	**KIF5A**	1.713	0.0008
H80240	IL8	1.7026	0.0008
R87126	LOC440918	1.6803	0.0008
RBM9	R87126	1.5856	0.0011
**SRF**	**DES**	1.5753	0.0011
R39465	TPM1	1.5546	0.0011
**PLAUR**	**MORF4L2**	1.5529	0.0011
**RPL30**	**IFITM2**	1.5288	0.0011
**IFITM2**	**DES**	1.5288	0.0011
**RPL30**	**CD37**	1.4324	0.0012
**RBM9**	**EIF2S2**	1.3979	0.0014
**TNNC1**	**IL8**	1.3532	0.0016
R39465	TNNC1	1.3325	0.0018
**TPT1**	**RPL30**	1.3308	0.0018
R39465	EPHB3	1.3291	0.0018
**RPS29**	**IL8**	1.3291	0.0018
R02593	MORF4L2	1.3118	0.0018
DES	RY1	1.2981	0.0018
**RPLP1**	**IL8**	1.2929	0.0019
R39465	NEUGRIN	1.2809	0.0021
**NK4**	**EPHB4**	1.2671	0.0023
T72863	IL8	1.243	0.0023
**EEF1G**	**IL8**	1.2378	0.0023
H87344	IL8	1.2292	0.0023
*** RPS9**	**DES**	1.2189	0.0023
**RBM9**	**ENO1**	1.2086	0.0023
HNRPA1	RY1	1.1776	0.0024
**RPL37**	**IL8**	1.1517	0.0024
R39465	MYL9	1.1483	0.0024
**TPT1**	**MORF4L2**	1.1466	0.0024
R39465	MEP50	1.1362	0.0025
RPS29	C15orf15	1.1294	0.0025
RPL30	H40095	1.0966	0.0026
**CANX**	**IL8**	1.0657	0.0026
**RPS29**	**AP3B2**	1.0639	0.0026
**OPHN1**	**IL8**	1.0588	0.0026
R39465	T72863	1.0553	0.0026
R39465	RY1	1.0519	0.0026
**NK4**	**EEF1G**	1.0519	0.0026
**RPS9**	**MORF4L2**	1.033	0.0029
**RPS29**	**RPL30**	1.0278	0.0029
**RPS29**	**ENO1**	0.9985	0.0032
**RPL30**	**AP3B2**	0.9968	0.0032
**RBM9**	**KIF5A**	0.9968	0.0032
DES	MEP50	0.9779	0.0032
**IL8**	**ATF4**	0.971	0.0033
R39465	PRPS1	0.9434	0.0035
R78934	EPHB4	0.9417	0.0035

**MT1G**	**IL8**	0.9331	0.0036
**BCL3**	**HNRPA1**	0.9297	0.0036
BCL3	RY1	0.9297	0.0036
TPM1	RY1	0.9056	0.0036
T74906	EIF2S2	0.9021	0.0036
T74906	IL8	0.9021	0.0036
MAOB	RY1	0.897	0.0036
TMSB4X	MEP50	0.8849	0.0036
**NK4**	**FUT1**	0.8849	0.0036
R39465	IFITM2	0.8763	0.0036
R39465	OPHN1	0.8728	0.0036
**RPL30**	**EPHB3**	0.8728	0.0036
*** DES**	**RPS9**	0.8677	0.0036
H73908	IL8	0.8608	0.0036
**A2M**	**IL8**	0.8591	0.0037
R39465	R39465	0.8556	0.0037
**NFIA**	**IL8**	0.8401	0.0037
**ENO1**	**DES**	0.8264	0.0039
**DES**	**HNRPD**	0.8264	0.0039
NEUGRIN	PRPS1	0.8264	0.0039
**DES**	**PHKG2**	0.8212	0.0041
R39465	BCL3	0.8057	0.0041
R39465	HNRPA1	0.8057	0.0041
**ENO1**	**AP3B2**	0.7902	0.0045
**HLA-B**	**EIF2S2**	0.7833	0.0045
RPS3	R87126	0.7799	0.0045
**RPS29**	**HSPD1**	0.7782	0.0045
R39465	SRF	0.7764	0.0045
R87126	RY1	0.7678	0.0045
**RBM9**	**MAOB**	0.7678	0.0045
**ENO1**	**EPHB4**	0.7627	0.0046
**TPM1**	**EPHB4**	0.7609	0.0046
**RPL30**	**MAOB**	0.7403	0.0047
**ZFP36L1**	**IL8**	0.7317	0.0047
**RPS9**	**MYH9**	0.7231	0.0048
**SRF**	**IL8**	0.7231	0.0048
ZFP36L1	MEP50	0.7231	0.0048
**ENO1**	**MAOB**	0.7196	0.0048
**TNNC1**	**EPHB4**	0.7179	0.0050
**ENO1**	**TPM1**	0.7162	0.0050
**PRTN3**	**IL8**	0.7093	0.0050
**RPL30**	**FGFR2**	0.699	0.0051
**DES**	**ACTA1**	0.6972	0.0051
**RPL30**	**KIF5A**	0.6938	0.0051
RPS9	R87126	0.6852	0.0054
R39465	UBTF	0.6749	0.0060
**EPHB4**	**UBTF**	0.6749	0.0060
**TPM1**	**PRPS1**	0.6749	0.0060
**UBTF**	**IL8**	0.6749	0.0060
R87126	H40095	0.6714	0.0062
**EIF2S2**	**IL1R2**	0.6714	0.0062
C15orf15	IL8	0.6714	0.0062
T72863	R87126	0.6559	0.0063
RBM9	NEUGRIN	0.6525	0.0064
SRF	NEUGRIN	0.6525	0.0064
**ENO1**	**EPHB3**	0.6525	0.0064
**CPSF1**	**IL8**	0.6525	0.0064

**CPSF1**	**FUT1**	0.6525	0.0064
TPT1	R87126	0.6353	0.0072
MEP50	H64807	0.6353	0.0072
**MORF4L2**	**MYL9**	0.6301	0.0073
R39465	MYL9	0.6267	0.0074
ACTB	MEP50	0.6198	0.0074
**RBM9**	**PHKG2**	0.6129	0.0074
**ACTB**	**ENO1**	0.6094	0.0074
**ENO1**	**EIF2S2**	0.6094	0.0074
**RPL30**	**MYH9**	0.6043	0.0074
**RPLP1**	**EIF2S2**	0.6043	0.0074
**RBM9**	**NFIA**	0.594	0.0074
**RBM9**	**EPHB3**	0.5888	0.0075
DES	C15orf15	0.5871	0.0077
T99080	IL8	0.5871	0.0077
**CHIT1**	**MYL9**	0.5853	0.0078
**ACTB**	**RPLP1**	0.5819	0.0080
R39465	EIF2S2	0.5819	0.0080
R02593	ENO1	0.5785	0.0081
**ENO1**	**MYL9**	0.5785	0.0081
**HLA-B**	**RPL37**	0.5698	0.0087
R87126	MEP50	0.5681	0.0087
H80240	MEP50	0.5664	0.0087
1108	C15orf15	0.5647	0.0087
**ENO1**	**FUT1**	0.5595	0.0090
T65938	NEUGRIN	0.5526	0.0093
R39465	T65938	0.5457	0.0097
RBM9	MEP50	0.544	0.0097
GYPA	R87126	0.5423	0.0097
R39465	CSRP1	0.5371	0.0098
CSRP1	NEUGRIN	0.5371	0.0098
SRF	H73908	0.5371	0.0098
**TMSB4X**	**IL8**	0.5371	0.0098

We only focused on the 52 genes whose functions are well characterized and documented in Gene Ontology. There are 109 significant gene-gene interactions in bold type among these 52 known genes. ‘*’ indicates that the two interactions are same because the two probes correspond to the same gene, RPS9.
