# Supplementary material for: Constructing disease-specific gene networks using pair-wise relevance metric: Application to colon cancer identifies interleukin 8, desmin and enolase 1 as the central elements
Source: BMC Syst Biol. 2008 Aug 10;2:72. doi: 10.1186/1752-0509-2-72 (PMC2535780; doi:10.1186/1752-0509-2-72)
Supplement: Additional file 2 — Application of the novel network approach to prostate cancer microarray data. [file 1752-0509-2-72-S2.pdf]

Application of the novel network approach to prostate cancer microarray data

In order to evaluate the power of our proposed network approach, it was applied to analyze another microarray data, prostate cancer data. We also obtained some significant results. Four hub genes in prostate cancer specific gene network, ANGPT1, CAV2, HPN and SLCO3A1, were proved to be responsible for prostate cancer pathogenesis. Two novel hub genes, ZNF532 and ID4, might define new central elements for prostate cancer. Gene ontology based analysis of gene-gene interactions and three-way gene interactions suggested that the tumorigenesis in prostate cancer was highly related to 'fatty acid oxidation' cell process and the categories associated with plasma membrance, which are well supported by multiple lines of experimental evidence.

Description of the prostate cancer data

The proposed approach was applied to a prostate cancer data, available from Lapointe et al. [1]. The Microarray data was pre-processed by the following steps: (1) carrying out base two logarithmic transformations; and (2) using normalization to have median 0 and standard deviation 1 per array. We deleted the clones that missing rate was larger than 20% in all experiments and the remaining missing values were replaced by using KNN impute imputation algorithm (k=15). Next, we mapped the remaining clones to gene symbols through SMD SOURCE tool [2]. Finally, we obtained 13236 gene expressions in 103 tissue samples (62 prostate cancer and 41 normal tissues).

Construction of prostate cancer specific gene network

5-fold cross validation resampling strategy was used to construct multiple replicates of training sets and test sets. By repeated this procedure 20 times, we obtained 500 pairs of data. On each pair, a classification tree was constructed and tested. In this way, a gene forest with 500 trees was constructed. From this gene forest, we identified 590 gene pairs (involving 200 genes) appearing in the same tree. The *AFV*s for these gene pairs ranged from 0.1294 to 9.6984. In order to determine their statistical significance, permutation technique was employed. Based on the estimated empirical null distribution of *AFV* obtained from estimating 17049 gene pairs in 1000 random trees, the threshold for significance level of 0.01 was 0.38. Thus, the gene pairs with *AFV* over the threshold were considered as having significant gene-gene interactions. We found 164 significant prostate cancer specific gene-gene interactions among 76 genes (as shown in Table 1). By connecting two genes in each gene pair, we constructed a gene network for prostate cancer (Figure 1).

Table 1. The *AFV* values of the 164 prostate cancer specific gene interactions

Gene	Gene	*AFV*	*p* value	Gene	Gene	*AFV*	*p* value
FLJ37644	ANGPT1	9.6984	0.0001	FLJ37644	DOCK3	0.63731	0.0033
SKAP1	ANGPT1	9.1255	0.0001	DOCK3	ANGPT1	0.63731	0.0033
ID4	HPN	8.9103	0.0001	SSTR1	ANGPT1	0.63619	0.0033
SLCO3A1	ANGPT1	8.9026	0.0001	ANGPT1	FLJ40243	0.63507	0.0033
WDR69	ANGPT1	7.5819	0.0001	LOC728176	HPN	0.62657	0.0036
LEPREL1	HPN	7.3346	0.0001	KIF13A	ANGPT1	0.62443	0.0036

WDR27	ANGPT1	7.0291	0.0001	WDR69	ZNF532	0.61755	0.0038
C9orf144	CAV2	6.1312	0.0001	LOC728176	ANGPT1	0.61705	0.0038
LOC161527	ANGPT1	5.5393	0.0001	FLJ37644	C7orf31	0.61689	0.0038
ZNF532	ANGPT1	4.5963	0.0001	C9orf144	FGFR2	0.61603	0.0038
C4orf35	CAV2	4.2819	0.0001	CAV2	ZNF532	0.60518	0.0039
CLGN	ANGPT1	4.1816	0.0001	WDR69	ELAC1	0.60315	0.0039
FLJ37644	SKAP1	3.6866	0.0002	LOC161527	ELAC1	0.60315	0.0039
WDR69	SKAP1	3.6418	0.0002	C7orf31	CRYAB	0.60147	0.0040
CLGN	WDR27	3.3108	0.0002	MCOLN3	CRYAB	0.60147	0.0040
C7orf31	ANGPT1	2.8196	0.0002	SUHW4	ANGPT1	0.58971	0.0043
ID4	LEPREL1	2.71	0.0002	PPP1R1B	CRYAB	0.58803	0.0043
COL5A3	ANGPT1	2.2037	0.0004	SEC24D	HPN	0.58243	0.0043
LPHN2	HPN	2.0482	0.0004	CDC27	ANGPT1	0.57795	0.0043
FLJ37644	KIAA0802	2.0326	0.0004	TMCO5	CAV2	0.55498	0.0047
ANGPT1	TSPAN15	1.9774	0.0004	FLJ37644	S100PBP	0.44853	0.0067
C7orf31	HPN	1.9767	0.0004	SLC14A1	LOC92345	0.44746	0.0067
DSCR1L2	HPN	1.9422	0.0004	SLCO3A1	ATP6V1H	0.4469	0.0067
FLJ37644	CAV2	1.8884	0.0004	C4orf35	MCOLN3	0.4357	0.0071
LOC92345	CAV2	1.8498	0.0006	C7orf31	FLJ40243	0.43514	0.0071
LOC161527	SKAP1	1.7348	0.0006	ID4	LOC728176	0.42664	0.0072
C4orf35	GJA1	1.6885	0.0006	SLCO3A1	LOC728176	0.42664	0.0072
TMCO5	ANGPT1	1.6493	0.0006	ID4	GBL	0.42664	0.0072
ELAC1	ANGPT1	1.6319	0.0007	GPRC5B	ZNF532	0.42664	0.0072
LOC728460	ANGPT1	1.4533	0.0007	SLCO3A1	ELAC1	0.42562	0.0072
S100PBP	ANGPT1	1.4463	0.0008	CAV2	NAT1	0.42562	0.0072
SLC4A3	ANGPT1	1.4275	0.0008	KIAA0802	PPARGC1A	0.42562	0.0072
C9orf144	GJA1	1.4264	0.0008	SLCO3A1	KIF13A	0.4245	0.0073
GBL	HPN	1.388	0.0008	LOC728460	FLJ40243	0.42338	0.0073
ID4	DSCR1L2	1.3306	0.0009	SLCO3A1	RNF32	0.4133	0.0074
NPEPL1	ANGPT1	1.3278	0.0009	GPT2	HPN	0.4133	0.0074
SIRPB1	PPARGC1A	1.3276	0.0009	SIRPB1	KIAA0802	0.41274	0.0075
FLJ37644	WDR69	1.3189	0.0009	LOC161527	COL5A3	0.41162	0.0075
MRPS31	ANGPT1	1.3104	0.0009	C4orf35	KIAA0802	0.41162	0.0075
FLJ37644	ZNF532	1.2986	0.0009	CAV2	PTPRO	0.40627	0.0079
TMCO5	WDR27	1.2477	0.0011	ID4	SEC24D	0.40322	0.0084
ARL5B	HPN	1.2135	0.0011	LOC728176	SLC14A1	0.40322	0.0084
FLJ37644	C4orf35	1.1917	0.0011	FLJ37644	PPARGC1A	0.40322	0.0084
C9orf144	HSPB8	1.1262	0.0012	SLC14A1	GJA1	0.40322	0.0084

FLJ37644	LOC161527	1.1184	0.0012	ARMC6	SLCO3A1	0.40307	0.0084
CAV2	MCOLN3	1.0751	0.0013	ARMC6	ANGPT1	0.40307	0.0084
ANGPT1	ATP6V1H	1.0735	0.0013	NFATC2IP	ANGPT1	0.4021	0.0086
SLC14A1	CAV2	1.073	0.0013	ZNF532	GJA1	0.4021	0.0086
C7orf31	MCOLN3	1.0393	0.0013	C9orf144	LOC728176	0.40154	0.0086
C9orf144	ZNF532	1.0314	0.0013	LOC161527	SUHW4	0.40154	0.0086
GPT2	ANGPT1	1.0073	0.0015	FLJ37644	CDC27	0.40154	0.0086
C7orf31	LEPREL1	0.96996	0.0016	WDR69	GPT2	0.40154	0.0086
FLJ37644	TSPAN15	0.90668	0.0016	CAV2	USP37	0.40154	0.0086
SLCO3A1	SKAP1	0.89548	0.0016	C4orf35	LOC92345	0.39986	0.0087
LPHN2	LEPREL1	0.8714	0.0016	FLJ37644	COL5A3	0.39986	0.0087
FLJ37644	C9orf144	0.85124	0.0017	BCAS1	HPN	0.39986	0.0087
ID4	ACOX1	0.85114	0.0017	ID4	LOC283378	0.39986	0.0087
ACOX1	HPN	0.85114	0.0017	HPN	LOC283378	0.39986	0.0087
SLCO3A1	SLC4A3	0.84676	0.0017	LOC161527	GPT2	0.39405	0.0091
NPAL3	ANGPT1	0.82649	0.0019	LEPREL1	ANGPT1	0.39237	0.0093
C9orf144	LOC92345	0.82604	0.0019	C9orf144	HOXB6	0.39034	0.0093
ID4	ARL5B	0.81361	0.0019	HOXB6	CAV2	0.39034	0.0093
CAV2	KIAA0802	0.81316	0.0019	C4orf35	ZNF532	0.39034	0.0093
C7orf31	CAV2	0.80669	0.0021	CAV2	AP3M2	0.39034	0.0093
MCOLN3	ANGPT1	0.80628	0.0021	CAV2	CEPT1	0.38978	0.0093
RNF32	ANGPT1	0.80364	0.0021	TCF15	ANGPT1	0.38978	0.0093
LOC728176	GJA1	0.79524	0.0022	M-RIP	GJA1	0.38978	0.0093
C7orf31	SKAP1	0.78795	0.0022	DPH4	GPR160	0.3881	0.0095
EMP2	ANGPT1	0.76932	0.0023	ACOT6	ANGPT1	0.3881	0.0095
LPHN2	TACSTD1	0.73307	0.0026	CYLC2	ANGPT1	0.3881	0.0095
CLGN	TSPAN15	0.68267	0.0028	ZNF587	GJA1	0.3881	0.0095
S100PBP	MRPS31	0.67198	0.0029	C7orf31	ID4	0.38285	0.0097
SLCO3A1	WDR27	0.67147	0.0029	LPHN2	PPARGC1A	0.38285	0.0097
FLJ37644	MRPS31	0.66134	0.0032	C7orf31	MOBK1B	0.38234	0.0098
FLJ37644	UFD1L	0.66083	0.0032	C7orf31	HSPB8	0.38234	0.0098
CAV2	UFD1L	0.66083	0.0032	MOBK1B	HSPB8	0.38234	0.0098
SLCO3A1	ZNF532	0.66027	0.0032	FLJ37644	ID4	0.38082	0.0098
LOC728460	MRPS31	0.64907	0.0032	C4orf35	LOC728176	0.38082	0.0098
C7orf31	ZNF532	0.64897	0.0032	C9orf144	M-RIP	0.38082	0.0098
C9orf144	KIAA0802	0.63843	0.0033	C9orf144	WDFY3	0.38082	0.0098
SLCO3A1	NPAL3	0.63833	0.0033	CAV2	WDFY3	0.38082	0.0098
LOC161527	ZNF532	0.63731	0.0033	FLJ37644	HPN	0.38082	0.0098

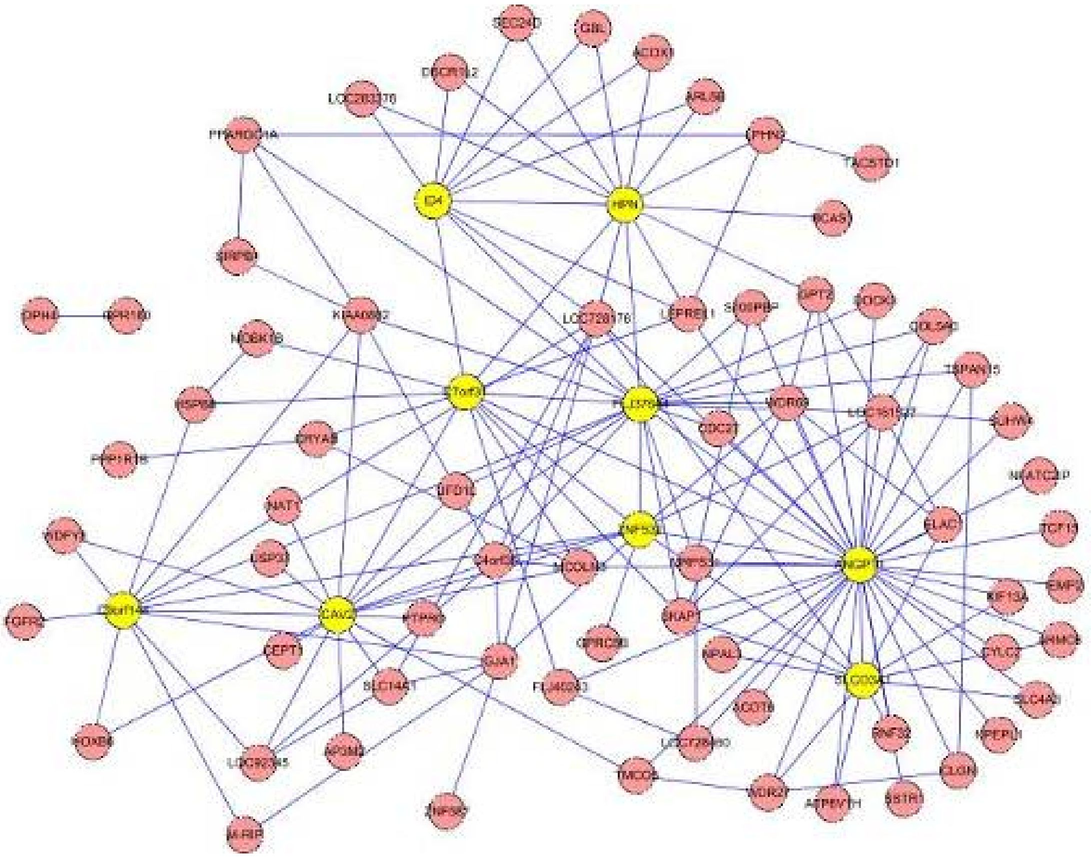

Figure 1. The prostate cancer specific gene network

In order to elucidate the functional implications of the built network, we used ‘Functional Annotation’ in DAVID Bioinformatics Resources to perform functional enrichment analysis based on Gene Ontology. The parameters were set as default. We identified 10 significant GO terms, as shown in Table 2. In order to identify more specific functions, we eliminated the redundant but broad ones among the 10 GO terms. Finally, we obtained 6 more specific GO terms (shown in bold type in Table 2). Based on the dimension of ‘Cellular Component’, we found that the pathogenesis of colon cancer was consistently linked to plasma membrane (integral to plasma membrane and intrinsic to plasma membrane). Prostate specific membrane antigen (PSMA) is a folate γ glutamyl carboxypeptidase that is oriented on the plasma membrane of normal and prostate cancer cells. As an integral membrane protein correlated with prostate cancer, PSMA offers a potentially valuable target for immunotherapy [3, 4]. Based on the dimension of ‘Biological Process’, we concluded that ‘response to nutrient levels’, ‘morphogenesis’, ‘fatty acid oxidation’ and ‘intracellular transport’ largely account for the prostate cancer tumorigenesis. These conclusions are well supported by multiple lines of experimental evidence. For example, recent studies showed that prostate cancer is associated with changes of fatty acid metabolism. Several enzymes involved in the metabolism of fatty acids have been determined to be altered in prostate cancer relative to normal prostate, which is indicative of an enhanced beta-oxidation pathway in prostate cancer. Liu demonstrated that dominant fatty acid metabolism rather than glycolysis has the potential to be the basis for imaging diagnosis and targeted treatment of prostate cancer [5].

Table 2. The GO terms significantly enriched with gene-gene interactions. In the bold style are the more specific GO terms

Category	GO term	*p*	Description
Biological Process	GO:0032502	0.036	development
	**GO:0031667**	**0.059**	**response to nutrient levels**
	GO:0009991	0.067	response to extracellular stimulus
	**GO:0009653**	**0.095**	**morphogenesis**
	**GO:0019395**	**0.096**	**fatty acid oxidation**
	**GO:0046907**	**0.097**	**intracellular transport**
Cellular Component	**GO:0005887**	**0.042**	**integral to plasma membrane**
	GO:0031226	0.043	intrinsic to plasma membrane
	GO:0005623	0.065	cell
Molecular Function	**GO:0051082**	**0.095**	**unfolded protein binding**

Identification of hub prostate cancer genes

We used a Poisson distribution to identify statistically significant hub nodes in prostate cancer specific network. At the significance level 0.05, we identified 9 hub genes: Angiopoietin-1 (ANGPT1, 37 connections, $p=1.11\times10^{-16}$), Hypothetical gene supported by AK094963 (FLJ37644, 20 connections, $p=4.27\times10^{-8}$), Caveolin2 (CAV2, 18 connections, $p=8.72\times10^{-7}$), Hepsin (HPN, 14 connections, $p=1.89\times10^{-4}$), Chromosome 7 open reading frame 31 (C7orf31, 13 connections, $p=6.23\times10^{-4}$), Solute carrier organic anion transporter family, member 3A1 (SLCO3A1, 12 connections, $p=0.0019$), Chromosome 9 open reading frame 144 (C9orf144, 12 connections, $p=0.0019$), Zinc finger protein 532 (ZNF532, 11 connections, $p=0.0054$), Inhibitor of DNA binding 4 (ID4, 11 connections, $p=0.0054$). Six known genes of the nine hub genes (ANGPT1, CAV2, HPN, SLCO3A1, ZNF532 and ID4) are all proved cancer-related hub genes. The evidences obtained by literature searching were as followings:

ANGPT1: Angiogenesis is essential for tumor growth and metastasis, and is coordinated by several classes of growth factors mediating their effect through receptors linked, in turn, to tyrosine kinase. Abnormal levels of Ang-1, Ang-2 and their receptor, Tie-2, are present in breast and prostate cancer, and their interrelationships may be important in the pathophysiology of these conditions [6]. In glandular prostate carcinoma, most of the tumor and intraglandular stromal cells were positive for both angiopoietin-1 and angiopoietin-2. Angiopoietin-1 and angiopoietin-2 were also found in tumor capillaries [7].

CAV2: Chene et al. found that CAV1, CAV2, MET and TES mRNA expression was lower in prostate tumors than in normal prostate tissues [8]. More importantly, Sowa et al. showed that caveolin-2 is phosphorylated in vivo at two serine residues and that the phosphorylation of caveolin-2 is necessary for its actions as a positive regulator of caveolin-1 during organelle biogenesis in prostate cancer cells [9]. The coding SNP in CAV2 in the total sample is associated with prostate cancer. The amino acid that is variable in CAV2 is Glu 130 Gln [10].

HPN: Pal et al. studied the association of 11 single nucleotide polymorphisms (SNPs) in the HEPSIN gene (HPN) with prostate cancer in men of European ancestry. HPN is a likely candidate in prostate cancer susceptibility, as it encodes a transmembrane cell surface serum protease, which is overexpressed in prostate cancer. A major 11-locus haplotype is significantly associated, which

provides further support that HPN is a potentially important candidate gene involved in prostate cancer susceptibility. Association of one of the SNPs with Gleason score is also suggestive of a plausible role of HPN in tumor aggressiveness [11].

HPN, has been identified as a marker gene of prostate cancer in recent studies [12-15]. HPN encodes hepsin, a cell surface transmembrane serine protease which plays an essential role in cell growth and maintenance of cell morphology. Using both cDNA and oligonucleotide microarray technologies, hepsin was shown to be significantly over-expressed in prostate cancer samples versus normal samples, and it has been identified as a potential biomarker for screening prostate cancer [14-17]. mRNA over-expression has also been validated using RT–PCR [15] and protein over-expression has been verified using tissue microarrays [14]. Magee et al. [17] also confirmed the over-expression of hepsin in prostate tumor by using the in situ hybridization technique on an independent panel of prostate specimens. Furthermore, the expression of hepsin has been shown to have positive correlation with prostate cancer staging [16], and to promote prostate cancer progression and metastasis [13]. Thus, hepsin may be used as a diagnostic as well as prognostic marker for prostate cancer [18].

SLCO3A1: Compared with normal liver, the expression of SLCO1B1, SLCO3A1, and SLCO1B3 was greatly repressed in tumoral (HepG2) cells, whereas SLCO2B1, SLC22A7, and SLC22A8 expression was either maintained or increased [19].

ZNF532: ZNF532 was identified as the robust progression signature whose expression decreased during the progression from benign epithelium to prostatic intraepithelial neoplasia (PIN) to prostate cancer to metastatic prostate cancer [20]. ZNF532 was identified as differentially expressed gene by using integrative analysis of genomic and transcriptomic profiles associated with prostate cancer progression [21].

ID4: Id (inhibitor of DNA binding) 4 is a member of the Id family of proteins (Id1-Id4), which function as dominant-negative regulators of basic helix-loop-helix transcription factors. Id factors are involved in numerous cell processes, including cell proliferation, differentiation, and tumorigenesis [22]. A major cause of treatment failure for prostate cancer is the development of androgen-independent metastatic disease. Id protein family, a group of basic helix–loop–helix transcription factors, has been shown to be involved in carcinogenesis and a prognostic marker in several types of human cancers. Yuen et al. suggest that in prostate cancer patients, differential Id proteins expressions may be a useful marker for poor prognosis, and Id-4 may be a potential prognostic marker for distant metastasis [23].

Pathway analysis of hub prostate cancer genes

To validate the newly identified 6 hub genes, we performed a pathway analysis using PathwayAssist software. We constructed the knowledge-based gene network (Figure 2) involving all cellular processes directly linked to these hub genes. These processes included differentiation, apoptosis, inflammation and pathogenesis etc. that are known to be pivotal for tumourigenesis. Based on this analysis, ANGPT1, CAV2, HPN and SLCO3A1 are proved central elements in this network. However, ZNF532 and ID4 are isolated points, which indicate that there is no any knowledge to prove their hub roles, as revealed by the above *AFV*-based networking, and these two genes may define new central elements in the prostate cancer specific gene network. We also conducted a pathway analysis to identify all cellular processes that link the 6 hub genes by implementing “Find all shortest paths between selected entities” in PathwayAssist Software.

Again, ANGPT1, CAV2, HPN and SLCO3A1 were the central elements (Figure 3). The common cellular processes for the four hub genes of ANGPT1, CAV2, HPN and SLCO3A1 greatly varied from proliferation, focal contact, endocytosis, assemble and mobility, due to the large number of cellular functions that ANGPT1 were linked.

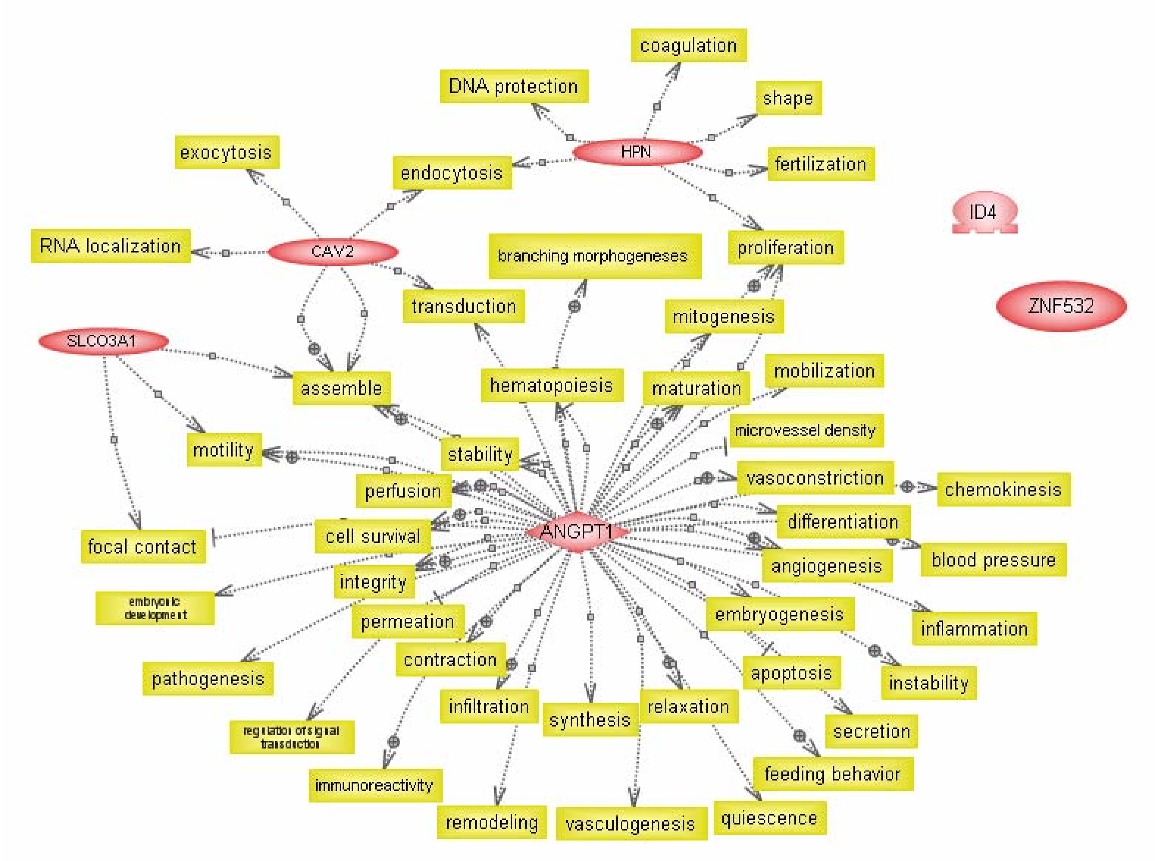

Figure 2. The knowledge-based gene network involving all cellular processes directly linked to the hub genes

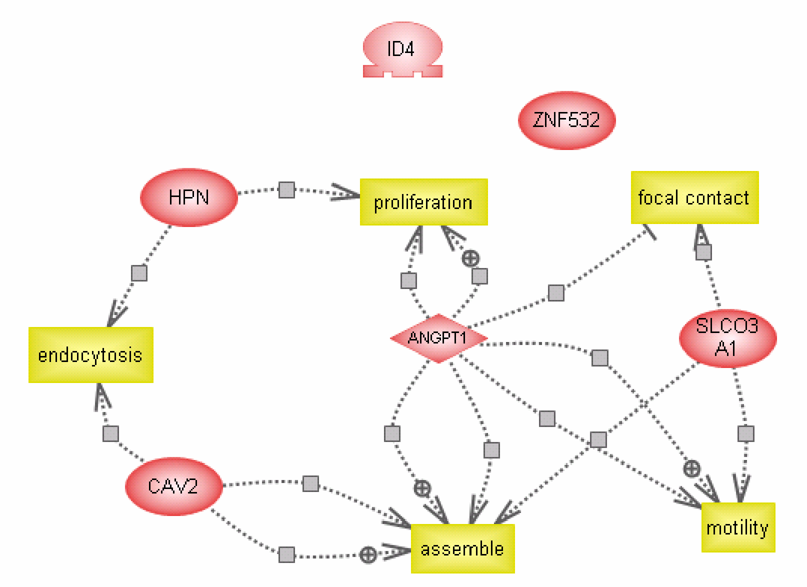

Figure 3. The knowledge-based gene network involving all cellular processes that linked among the hub genes

High-order interactions in the prostate cancer specific gene network

In the prostate cancer specific gene network, 98 three-way interactions (triangles) among 56 genes were identified. Based on 1000 random networks, the triangle was significantly over-represented (p=0.04) in this network at the significance level 0.05. Then, an enrichment analysis based on GO was performed using default parameters by DAVID resources. We identified 13 GO functional categories (Table 3). These results were consistent with the enrichment analysis of two-way interactions, suggesting that the above categories largely captured the functional facets of the prostate cancer specific gene network.

Table 3. The GO terms significantly enriched with three-way interactions. In the bold style are the more specific GO terms

Category	GO term	p	Description
Biological Process	GO:0032502	0.043	development
	GO:0006810	0.055	transport
	**GO:0006811**	**0.056**	**ion transport**
	**GO:0009887**	**0.059**	**organ morphogenesis**
	**GO:0019395**	**0.061**	**fatty acid oxidation**
	**GO:0048513**	**0.072**	**organ development**
	**GO:0051649**	**0.085**	**establishment of cellular localization**
	GO:0051179	0.087	localization
	**GO:0006457**	**0.092**	**protein folding**
	GO:0016043	0.094	morphogenesis
Cellular Component	**GO:0005887**	**0.045**	**integral to plasma membrane**
	GO:0031226	0.046	intrinsic to plasma membrane
Molecular Function	**GO:0051082**	**0.053**	**unfolded protein binding**

References

1. Lapointe J, Li C, Higgins JP, van de Rijn M, Bair E, Montgomery K, Ferrari M, Egevad L, Rayford W, Bergerheim U *et al*: **Gene expression profiling identifies clinically relevant subtypes of prostate cancer**. *Proc Natl Acad Sci U S A* 2004, **101**(3):811-816.
2. Diehn M, Sherlock G, Binkley G, Jin H, Matese JC, Hernandez-Boussard T, Rees CA, Cherry JM, Botstein D, Brown PO *et al*: **SOURCE: a unified genomic resource of functional annotations, ontologies, and gene expression data**. *Nucleic Acids Res* 2003, **31**(1):219-223.
3. Anilkumar G, Barwe SP, Christiansen JJ, Rajasekaran SA, Kohn DB, Rajasekaran AK: **Association of prostate-specific membrane antigen with caveolin-1 and its caveolae-dependent internalization in microvascular endothelial cells: implications for targeting to tumor vasculature**. *Microvasc Res* 2006, **72**(1-2):54-61.
4. Schmittgen TD, Teske S, Vessella RL, True LD, Zakrajsek BA: **Expression of prostate specific membrane antigen and three alternatively spliced variants of PSMA in prostate cancer patients**. *Int J Cancer* 2003, **107**(2):323-329.
5. Liu Y: **Fatty acid oxidation is a dominant bioenergetic pathway in prostate cancer**. *Prostate Cancer Prostatic Dis* 2006, **9**(3):230-234.

6. Caine GJ, Blann AD, Stonelake PS, Ryan P, Lip GY: **Plasma angiopoietin-1, angiopoietin-2 and Tie-2 in breast and prostate cancer: a comparison with VEGF and Flt-1**. *Eur J Clin Invest* 2003, **33**(10):883-890.
7. Wurmbach JH, Hammerer P, Sevinc S, Huland H, Ergun S: **The expression of angiopoietins and their receptor Tie-2 in human prostate carcinoma**. *Anticancer Res* 2000, **20**(6D):5217-5220.
8. Chene L, Giroud C, Desgrandchamps F, Boccon-Gibod L, Cussenot O, Berthon P, Latil A: **Extensive analysis of the 7q31 region in human prostate tumors supports TES as the best candidate tumor suppressor gene**. *Int J Cancer* 2004, **111**(5):798-804.
9. Sowa G, Pypaert M, Fulton D, Sessa WC: **The phosphorylation of caveolin-2 on serines 23 and 36 modulates caveolin-1-dependent caveolae formation**. *Proc Natl Acad Sci U S A* 2003, **100**(11):6511-6516.
10. Burmester JK, Suarez BK, Lin JH, Jin CH, Miller RD, Zhang KQ, Salzman SA, Reding DJ, Catalona WJ: **Analysis of candidate genes for prostate cancer**. *Hum Hered* 2004, **57**(4):172-178.
11. Pal P, Xi H, Kaushal R, Sun G, Jin CH, Jin L, Suarez BK, Catalona WJ, Deka R: **Variants in the HEPSIN gene are associated with prostate cancer in men of European origin**. *Hum Genet* 2006, **120**(2):187-192.
12. Nelson PS: **Predicting prostate cancer behavior using transcript profiles**. *J Urol* 2004, **172**(5 Pt 2):S28-32; discussion S33.
13. Klezovitch O, Chevillet J, Mirosevich J, Roberts RL, Matusik RJ, Vasioukhin V: **Hepsin promotes prostate cancer progression and metastasis**. *Cancer Cell* 2004, **6**(2):185-195.
14. Dhanasekaran SM, Barrette TR, Ghosh D, Shah R, Varambally S, Kurachi K, Pienta KJ, Rubin MA, Chinnaiyan AM: **Delineation of prognostic biomarkers in prostate cancer**. *Nature* 2001, **412**(6849):822-826.
15. Luo J, Duggan DJ, Chen Y, Sauvageot J, Ewing CM, Bittner ML, Trent JM, Isaacs WB: **Human prostate cancer and benign prostatic hyperplasia: molecular dissection by gene expression profiling**. *Cancer Res* 2001, **61**(12):4683-4688.
16. Stamey TA, Warrington JA, Caldwell MC, Chen Z, Fan Z, Mahadevappa M, McNeal JE, Nolley R, Zhang Z: **Molecular genetic profiling of Gleason grade 4/5 prostate cancers compared to benign prostatic hyperplasia**. *J Urol* 2001, **166**(6):2171-2177.
17. Magee JA, Araki T, Patil S, Ehrig T, True L, Humphrey PA, Catalona WJ, Watson MA, Milbrandt J: **Expression profiling reveals hepsin overexpression in prostate cancer**. *Cancer Res* 2001, **61**(15):5692-5696.
18. Xu L, Tan AC, Naiman DQ, Geman D, Winslow RL: **Robust prostate cancer marker genes emerge from direct integration of inter-study microarray data**. *Bioinformatics* 2005, **21**(20):3905-3911.
19. Libra A, Fernetti C, Lorusso V, Visigalli M, Anelli PL, Staud F, Tiribelli C, Pascolo L: **Molecular determinants in the transport of a bile acid-derived diagnostic agent in tumoral and nontumoral cell lines of human liver**. *J Pharmacol Exp Ther* 2006, **319**(2):809-817.
20. Tomlins SA, Mehra R, Rhodes DR, Cao X, Wang L, Dhanasekaran SM, Kalyana-Sundaram S, Wei JT, Rubin MA, Pienta KJ *et al*: **Integrative molecular concept modeling of prostate cancer progression**. *Nat Genet* 2007, **39**(1):41-51.

21. Kim JH, Dhanasekaran SM, Mehra R, Tomlins SA, Gu W, Yu J, Kumar-Sinha C, Cao X, Dash A, Wang L *et al*: **Integrative analysis of genomic aberrations associated with prostate cancer progression**. *Cancer Res* 2007, **67**(17):8229-8239.
22. de Candia P, Akram M, Benezra R, Brogi E: **Id4 messenger RNA and estrogen receptor expression: inverse correlation in human normal breast epithelium and carcinoma**. *Hum Pathol* 2006, **37**(8):1032-1041.
23. Yuen HF, Chua CW, Chan YP, Wong YC, Wang X, Chan KW: **Id proteins expression in prostate cancer: high-level expression of Id-4 in primary prostate cancer is associated with development of metastases**. *Mod Pathol* 2006, **19**(7):931-941.
